# Transcriptome profiling of Stevia rebaudiana MS007 revealed genes involved in flower development

**DOI:** 10.3906/biy-2103-3

**Published:** 2021-06-23

**Authors:** Nurul Hidayah SAMSULRIZAL, Khairul Shahyidi KHADZRAN, Tamil Chelvan MEENAKSHI SUNDRAM, Zarina ZAINUDDIN, Siti Hajar Nor SHAARANI, Nur Sabrina Ahmad AZMI, Sarahani HARUN

**Affiliations:** 1 Department of Plant Science, Kulliyyah of Science, International Islamic University Malaysia, Kuantan, Pahang Malaysia; 2 Centre for Bioinformatics Research, Institute of Systems Biology, National University of Malaysia , Bangi, Selangor Malaysia; 3 Faculty of Chemical and Process Engineering Technology, Malaysia University Pahang Malaysia

**Keywords:** De novo transcriptome, Illumina, differentially expressed genes, *Stevia rebaudiana*, flowering genes

## Abstract

*Stevia rebaudiana* is a medicinal plant recommended to diabetic or obese patients as an alternative sweetener owing to its low-calorie property. Previous studies have found that the stevioside level is highest at the time of flower bud formation and lowest at the time of preceding and following flower bud formation. Hence, this study aims to identify the genes involved in the flowering of local *S. rebaudiana* accession MS007 by investigating the transcriptomic data of two stages of growth, before flowering (BF) and after flowering (AF) that were deposited under accession number SRX6362785 and SRX6362784 at the NCBI SRA database. The transcriptomic study managed to annotate 108299 unigenes of *S. rebaudiana* with 8871 and 9832 genes that were differentially expressed in BF and AF samples, respectively. These genes involved in various metabolic pathways related to flower development, response to stimulus as well as photosynthesis. Pheophorbide A oxygenase ( *PAO* ), eukaryotic translation initiation factor 3 subunit E ( *TIF3E1* ), and jasmonate ZIM domain-containing protein 1 ( *JAZ1* ) were found to be involved in the flower development. The outcome of this study will help further research in the manipulation of the flowering process, especially in the breeding programme to develop photo-insensitive *Stevia* plant.

## 1. Introduction

*Diabetes mellitus* is one of the critical diseases in the past 3000 years since the ancient Egyptian civilization (Lakhtakia, 2013). According to the research by Stanford Prevention Research Centre, sugar appears to contribute to the risk of diabetes besides calorie and total calorie intake. As an alternative, *Stevia* plants are currently used as a sweetener to replace sugar. *Stevia rebaudiana* powdered leaf has been used for over a century in Paraguay to sweeten beverages. Although this cultural practice is done in a small scale, larger quantities of *S. rebaudiana* have increasingly been used in the last quarter of a century in Japan as more beverages containing stevioside are now available (Kinghorn, 2002).The commercialization of *S. rebaudiana* leaves for sweetening and flavouring purposes has been on the rise since its introduction in Japan. The cultivation of *S. rebaudiana* for the Japanese market also occurs to some degree in China, Taiwan, Korea, Thailand, and Indonesia (Prakash and Chaturvedula, 2016). Stevioside, the primary compound of steviol glycosides, was first isolated by two French chemists, Bridel and Lavielle in 1931 from the *Stevia* leaves (Brandle et al., 1998). 

Steviol glycosides are responsible for the distinct sweet taste of *S. rebaudiana* . However, several other compounds such as essential oils, tannins, and flavonoid in the *S. rebaudiana* crude extracts cause the off tastes that limit their commercial development (Prakash et al., 2008; Samuel et al., 2018). Thus, attempts were made to purify the extracts and chemically characterize the steviol glycosides (Prakash et al., 2008). The amount of steviol glycosides varies between different parts of the plant, with the highest content recorded in leaves (Karimi et al., 2014). Abdullateef and Osman (2011) reported that during crop production, growers could manipulate the length of the day to influence the flowering of crops sensitive to photoperiod by adding supplemental lighting to a crop, increasing the amount of photosynthesis and affecting plant growth (Abdullateef and Osman, 2011). *Stevia* requires short days for flowering with a critical day length of 13 h. *Stevia* plants are reported to flower earlier when treated with short days and will delay flowering if treated with long days (Runkle and Heins, 2001). Kim et al. (2002) recorded that the optimum harvesting time was determined by the leaf dry weight and stevioside content depending on the flowering period; they are highest at the time of flower bud formation and lowest at the time of preceding and following bud formation (Kim et al., 2002). 

However, there is a lack of evidence that shows the role of flowering genes in *S. rebaudiana* . The development of next-generation sequencing (NGS) methodologies has offered useful strategies to overcome this problem. Identification of genes involved in the flowering process will improve *Stevia* plant production to not only produce a high yield of leaves, but also allow development of photo-insensitive *Stevia* . The objective of this project is to generate and analyze the transcriptomic libraries of S *. rebaudiana* , and to identify the genes involved in the flowering process by using bioinformatics tools. The number of genes involved in the flowering process in *S. rebaudiana* was identified using bioinformatics approaches, including constructing, sequencing and analysing transcriptomic libraries.

## 2. Materials and methods

### 2.1. Sample and library preparation 

*S. rebaudiana* accession MS007 was cultivated through shoot cutting method to yield sufficient plant materials. A healthy *Stevia* accession MS007 mother plant was obtained from the nursery of Kulliyyah of Science, International Islamic University Malaysia. Healthy stems with at least 2 leaves were selected for stem propagation as the stem rooted easily (Yadav and Guleria, 2012). No additional chemical or organic fertilizer was added, and the plants were allowed to grow for 40 days for flowering to occur with 12–13 h exposure to daylight. In this study, two groups of samples were determined: 1) leaves collected one week before flowering (BF); 2) leaves collected after flowering (AF). For the Illumina library preparations, the total RNA was extracted from 100 mg of collected leaf tissues using the Geneaid Total RNA Mini Kit (Plant) (Geneaid Biotech Ltd, New Taipei City, Taiwan, Cat. #RP050) following the manufacturer’s protocol, and the libraries were prepared using the mRNA-Seq 2 sample prep kit (Illumina Inc. San Diego, CA, USA, Cat. RS-100-0801). 

### 2.2. Analysis of transcriptomic data

Quality control (QC) was performed at each step of the procedures to guarantee the reliability of the RNA-seq data. For the QC, base-calling accuracy was measured by the Phred quality score (Q score) (Richterich, 1998). The gene expression levels were determined by quantifying the observed read abundance. The original raw data from Illumina were transformed into sequenced reads by base-calling. The following quality symbol approaches were executed in which the data counts were estimated by re-mapping raw short reads to the assembled contigs using bowtie. The RNA-seq by expectation-maximization (RSEM) package (Li and Dewey, 2011) was used to resolve the ambiguous mapping and to perform final quantifications. Only paired-end reads that mapped to a common contig were considered. Normalization was done by calculating the RPKM values (read per kilobase of exon model per million mapped reads) for each contig. The raw sequence data of BF (before flowering sample) and AF (after flowering sample) were deposited in NCBI SRA database under accession number SRX6362785 and SRX6362784, respectively (Samsulrizal et al., 2020). Raw data that had passed the quality control were recorded in a FASTQ file, which contained the sequence information (reads) and corresponding sequencing quality information. 

### 2.3. Gene functional annotation

To achieve a comprehensive functional gene annotation, seven databases were applied in this study. The function and characteristics of the seven databases are as follows: 1) NCBI nonredundant protein sequences ( *nr* ): it is the formal protein sequence databases of NCBI, which includes protein sequence information from GenBank; 2) Protein Data Bank (PDB): it is the structural protein database; 3) Swiss-Prot: it is a curated protein sequence database; 4) Protein Information Resource (PIR): it is a protein sequence database; 5) Protein Research Foundation (PRF): it stores amino acids, peptides and proteins information; 6) NCBI nucleotide sequences ( *nt* ): it is the formal nucleotide sequence database of NCBI; and 7) RefSeq: it is a curated nonredundant database of genomes, transcripts, and proteins.

Pfam (protein family) is a manually curated collection of protein families available via the web and in flat file form (Sonnhammer et al., 1997). It is represented as multiple sequence alignments containing all members of the family as detected with a profile hidden Markov model (HMM). Through HMMER3 program, the established HMM model can be searched to annotate the unigenes (Punta et al., 2012). Meanwhile, both Cluster of Orthologous Groups of proteins (COG) and EuKaryotic Orthologous Groups (KOG) are based on NCBI’s gene orthologous relationships. COG is specific to prokaryotes while KOG is specific to eukaryotes (Tatusov et al., 2003). Gene Ontology (GO) is an established standard for the functional annotation of gene products. GO vocabulary is a controlled vocabulary used to classify the following functional attributes of gene products: biological process (BP), molecular function (MF) and cellular component (CC) that represents the cellular localization of gene product (Wang et al., 2004; Yon Rhee et al., 2008). 

### 2.4. Gene expression analysis and GO enrichment analysis

Gene expression analysis is used to calculate gene expression levels that connects the information encoded in a gene with the final functional gene product, such as a protein. For alignment references, de novo transcriptome filtered by Corset was used as a reference (Davidson and Oshlack, 2014). Corset is a method that hierarchically clusters contigs using shared reads and expression, and then summarizes read counts to clusters, ready for statistical testing (Davidson and Oshlack, 2014). RSEM package (Li and Dewey, 2011) would map reads back to the transcriptome and quantify the expression level. The expression data were organized, and the heat map was generated using ClustVis (Metsalu and Vilo, 2015). The GO enrichment analysis was performed using BiNGO by undergoing detailed identification of overrepresented GO terms in the provided genes in the study (Maere et al., 2005).

## 3. Results

### 3.1. Illumina sequencing and de novo sequence assembly

The error rate distribution of reads for BF and AF samples was minimum, which was more than 0.06% error at 150 position along the reads. The GC content distribution is evaluated to detect potential AT/GC separation, which affects subsequent gene expression quantification. The results of the GC distribution for both BF and AF showed equal distribution with AT type gene, proving no potential separation between AT/GC genes. Raw reads were filtered to remove reads containing adapters or low-quality reads; consequently, the filtering process involved elimination of reads with adaptor contamination (Dodt et al., 2012). 

The results from the BF sample contained 75.99% of clean reads, showing an improved average quality and reduced volume of erroneous data. This could include the removal of poor or biased sequence, ambiguous bases, read duplicates, homopolymers that formed at the edge of a flow cell and adapter dimers as adapter-related error was 19.79%. The low percentage of low-quality reads was significant, which was 0.68%. Meanwhile, the reads containing undefined nucleotides were only 3.54%. The results from the AF sample showed that it contained 82.84% of clean reads as the adapter-related error was 14.86%. The low percentage of low-quality reads was significant, which was 0.60%. Meanwhile, the reads containing undefined nucleotides were only at 1.70%. The summary of quality control results is shown in Table S1.

### 3.2. Gene functional annotation

To achieve a comprehensive functional gene annotation, several databases were applied in this study that include GeneOntology (GO), UniProt, GenBank, and Pfam. The statistical analysis of successfully annotated genes from both *Stevia* samples by each database is shown in Table S2. For the species distribution, 11.8% of the distinct sequences have top matches (first hit) trained with sequences from the Vitaceae family of grape ( *Vitis vinifera* ), followed by sesame plant ( *Sesamum indicum* ; 8.3%), robusta coffee ( *Coffea canephora* ; 7.2%), woodland tobacco ( *Nicotiana sylvestris* ; 4.9%), and wild tobacco ( *Nicotiana tomentosiformis* ; 4.4%). The *e* -value distribution of the top hits in the nr database showed that 24.1% of the mapped sequences had strong homology (smaller than 1.0E-100). The results for the similarity distribution in this showed that 43.9% of the sequences had a similarity between 60% and 80% while 35.4% of the hits had a similarity ranging from 80% to 90%.

Meanwhile, the sequence similarity searches can be identified as homologous proteins or genes by detecting excess similarity or statistically significant similarity that reflects common ancestry (Pearson, 2013). The results for the similarity distribution in this showed that 43.9% of the sequences had a similarity between 60% and 80% while 35.4% of the hits had a similarity ranging from 80% to 90%.

### 3.3. Gene expression analysis and analysis of differentially expressed gene

**Figure 1:**
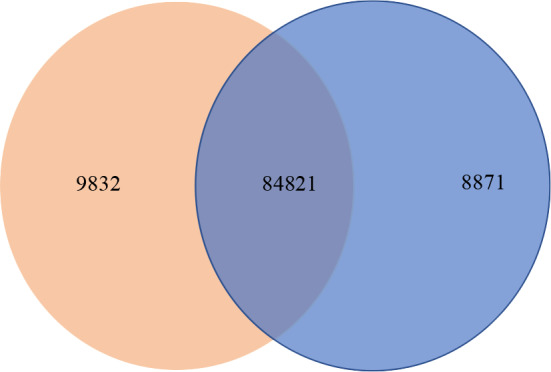
Venn diagram of gene expression in S. rebaudiana MS007 in two different samples (BF and AF).

For alignment references, de novo transcriptome filtered by Corset was used as a reference. Corsetis a method that hierarchically clusters contigs using shared reads and expression, and then summarizes read counts to clusters, ready for statistical testing (Davidson and Oshlack, 2014). RSEM package (Li and Dewey, 2011) would map reads back to the transcriptome and quantify the expression level. The summary of mapping results is shown in Table S3. For the expressed gene, those two groups of BF and AF samples are shown in the Venn diagram (Figure 1). Diagram below shows 9832 genes for AF and 8871 genes for BF, and the 84,821 overlapping genes represent the genes commonly expressed by both samples, using FPKM > 0.3 as the criterion.

**Figure 2:**
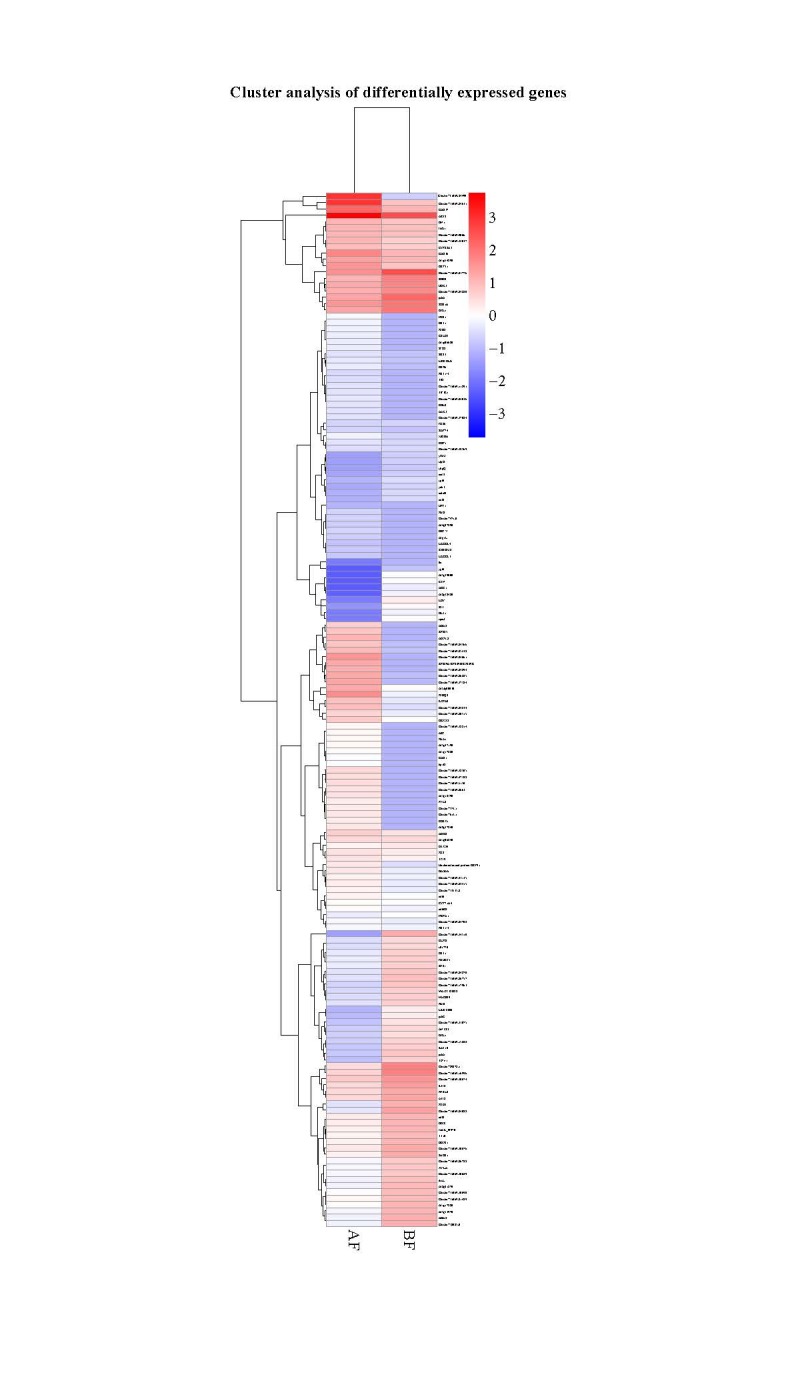
Differently expressed genes of the two samples. Stevia BF represents before flowering cluster of expressed genes and Stevia AF represents after flowering sample of S. rebaudiana MS007.

Gene expression analysis was conducted to determine the functions of the gene products. In this study, expressed flowering genes were found in both samples of differently expressed genes. Figure 2 shows the differently expressed genes, which consist of two samples. Both BF and AF samples of *S. rebaudiana* were compared against each other for different expression levels of genes. The red color represents upregulated genes and blue color represents downregulated genes. In this study, 108,299 unigenes were annotated, in which 8871 genes were differentially expressed in the BF sample, and 9832 genes were differentially expressed in the AF sample. The list of selected up-regulated and down-regulated genes in this study is shown in Table 1.

**Table 1:** List of selected differentially expressed genes in BF and AF samples of S. rebaudiana MS007.

Gene ID	log2fold change	UniProt ID	Gene name	Protein name
Upregulated genes				
Cluster-31069.33179	6.0235	Q9FGM1	PYL8	Abscisic acid receptor PYL8
Cluster-31069.23425	5.6635	Q9C5Z3	TIF3E1	Eukaryotic translation initiation factor 3 subunit E
Cluster-31069.29506	5.637	Q9FJR0	UPF1	Regulator of nonsense transcripts 1 homolog
Cluster-31069.16061	5.3188	Q9FYC2	PAO	Pheophorbide a oxygenase, chloroplastic
Cluster-15330.0	3.4635	Q6NMQ7	GASA6	Gibberellin-regulated protein 6
Cluster-17717.4	2.7135	Q9SSQ4	FRS6	Protein FAR1-RELATED SEQUENCE 6
Cluster-31069.3242	1.1024	Q9LMA8	JAZ1	Protein TIFY 10A (Jasmonate ZIM domain-containing protein 1)
Downregulated genes				
Cluster-31069.12927	–6.2222	O49255	NAC29	NAC transcription factor 29
Cluster-31069.23727	–4.504	Q6R0H1	LHY	Protein LHY
Cluster-31069.35616	–2.7784	P25818	TIP1-1	Aquaporin TIP1-1
Cluster-31069.33588	–2.3188	Q96289	ZAT10	Zinc finger protein ZAT10
Cluster-31069.36919	–1.7876	Q93WF6	SAG21	Protein SENESCENCE-ASSOCIATED GENE 21, mitochondrial
Cluster-31069.24565	–1.497	Q9FGY1	BXL1	Beta-D-xylosidase 1
Cluster-31069.30674	–1.436	P27140	BCA1	Beta carbonic anhydrase 1, chloroplastic
Cluster-31069.19662	–1.1932	P43297	RD21A	Cysteine proteinase RD21A
Cluster-31069.26728	–1.1447	P49078	ASN1	Asparagine synthetase
Cluster-31069.25045	–1.1245	Q84J37	TT10	Laccase-15
Cluster-31069.23317	–1.0374	Q8LDN8	UGE3	Bifunctional UDP-glucose 4-epimerase and UDP-xylose 4-epimerase 3
Cluster-31069.38678	–1.0277	Q9XI73	PNSL2	Photosynthetic NDH subunit of lumenal location 2, chloroplastic

### 3.4. GO enrichment analysis of differentially expressed genes

In this study, we found differentially expressed genes that are related to flowering which include seven upregulated genes: *PYL8, TIF3E1, UPF1, PAO, GASA6, FRS6* and *JAZ1* and twelve downregulated genes: *LHY, SAG21, ZAT10,TIP1-1, NAC29, BXL1, BCA1, RD21A, ASN1, TT10, UGE3,* and *PNSL2* (Table 1). The up-regulated genes were associated with significant biological processes such as cellular response to organic substance, response to sucrose stimulus, and flower development. The enriched GO that were enriched within the down-regulated genes were response to chemical stimulus, reproductive structure development, response to light intensity, response to gibberellin stimulus, photosynthesis, developmental process, and carbon utilization.

## 4. Discussion

The research aim of this study is to determine the role of flowering genes in *S. rebaudiana* by using NGS approach. Thus, the transcriptomic libraries of *S. rebaudiana* in two samples, BF (before flowering sample) and AF (after flowering sample) that were successfully deposited in NCBI SRA database were generated and analyzed. Bioinformatics techniques such as designing, sequencing, and analysing transcriptomic libraries were used to identify the genes involved in the flowering process in *S. rebaudiana* . Next, downstream transcriptome analysis such as GO enrichment approach was conducted to characterize the differentially expressed genes to infer the significant biological processes in the two samples.

There were seven genes found to be upregulated during the flowering: *PYL8, TIF3E1, UPF1, PAO, GASA6, FRS6* and *JAZ1* (Table 1). Cluster-31069.33179 or known as pyrabactin resistance-like protein 8 ( *PYL8* ) had the highest expression after the flowering process which was involved during cases of dehydration (Lim et al., 2013). It was followed by *TIF3E1* gene that encoded a heavily expressed protein in pollen seeds and growth of embryos and root tips, and interacted with the proteins of Arabidopsis: eIF3e and eIF3h. It was shown that pollen germination and embryo development were affected in the transgenic mutation of *TIF3E1* gene (Xia et al., 2010). Besides that, based on Table 1, twelve genes were downregulated after the flowering process in *S. rebaudiana* , such as *LHY, SAG21,* *ZAT10,* and *NAC29* . Late elongated hypocotyl ( *LHY* ) helps to regulate the circadian clock of the *S. rebaudiana* by not only repressing the floral transition under short-day and long-day conditions, but also accelerating flowering when the plants were grown under continuous light (Fujiwara et al., 2008). *Stevia* plant requires 13 h of photoperiod to regulate flowering process, thus proving that this gene is involved in the cascade of reaction for the flowering together with the photoperiod genes. These genes were found to be involved in various metabolic pathways related to flower development, response to stimulus as well as photosynthesis.

*SAG21* gene belongs to the late embryogenesis associated (LEA) protein family, which is also known as AtLEA 5 (late embryogenesis abundant like 5) (Tang et al., 2016). The functions of this gene have been discovered in *A. thaliana* , including to promote root development and prevent premature aging such as flowering and senescence (Salleh et al., 2012). Salleh et al. (2012) also reported that this gene was recognized at an early stage of leaf senescence when the leaves started to change color from green to yellow and when the plant was induced by darkness or absence of sunlight. The expression of the gene reduced once the leaves reached total senescence. Meanwhile, based on UniProt resources, the *ZAT10* gene functions as zinc finger for salt tolerance. Cys2/His2-type zinc finger protein found in *A. thaliana* has shown to function as a transcription repressor under drought, cold and high salinity stress condition (Sakamoto et al., 2004). 

**Figure 3:**
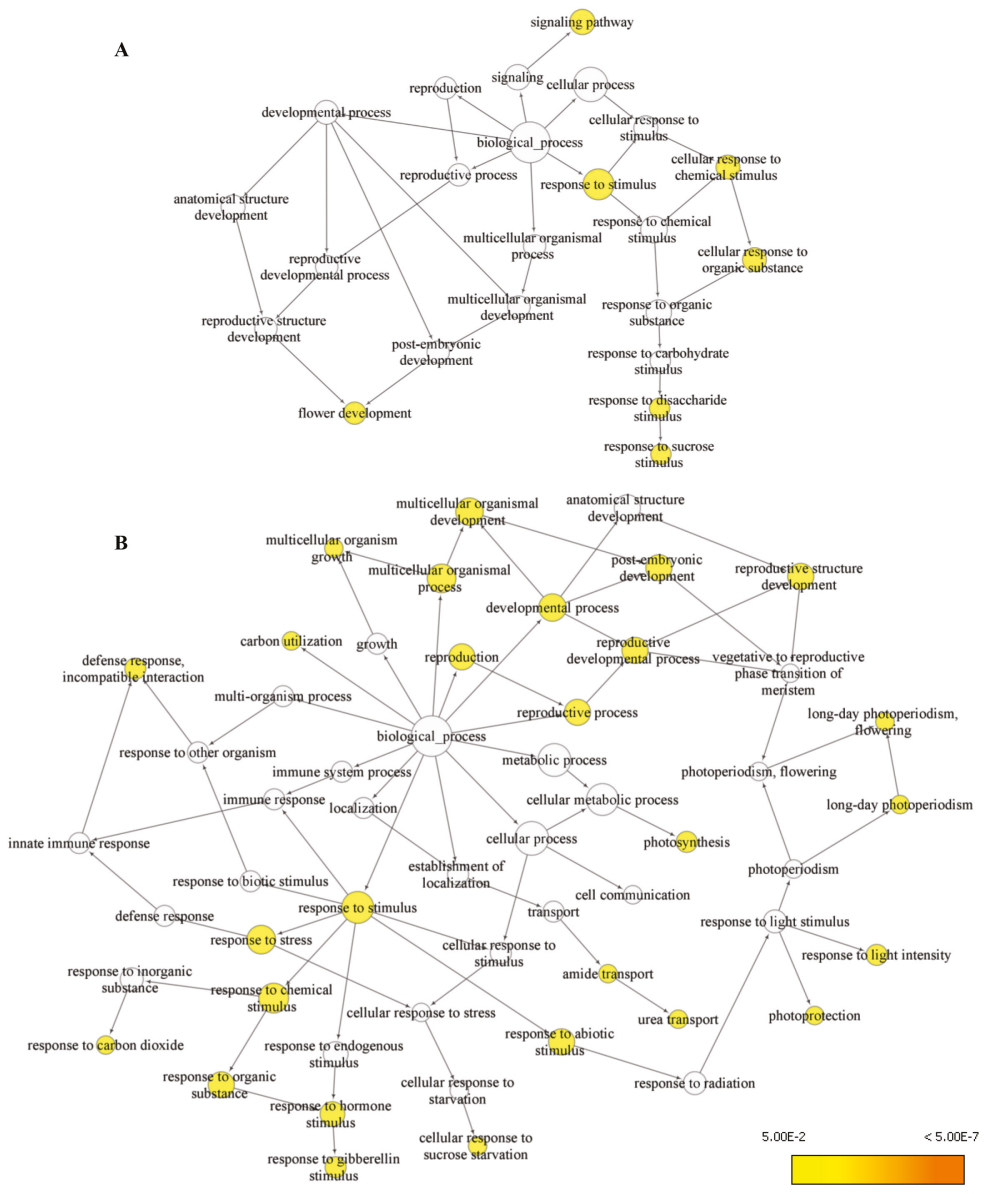
The overrepresented GO biological process in the differentially expressed genes. A) Overrepresented GO biological processes identified in upregulated genes. B) Overrepresented GO biological processes identified in downregulated genes.

Combined assembly related downstream studies, such as functional annotation and network analysis offer insights into the biological processes applicable in the leaf of *S. rebaudiana* . “Flower development” and “response to sucrose stimulus” have been recognised as significantly enriched in the biological process of *S. rebaudiana* (Figure 3). Based on the abundant transcripts and GO enrichment analysis in *S. rebaudiana* leaf before and after flowering (Table 2), we focused on identifying all the transcripts related to the flower development biosynthesis. In general, the genes related to flower development biosynthesis were *PAO* , *TIF3E1* , and *JAZ1* with significant *p* -values.

**Table 2:** The list of significantly enriched GO terms of S. rebaudiana MS007 identified from the BiNGO app.

GO ID	Description	Gene list	p-value
Upregulated genes			
71310	Cellular response to organic substance	PYL8, UPF1, GASA6, JAZ1	3.0764E-4
9744	Response to sucrose stimulus	UPF1, GASA6	5.9293E-4
9908	Flower development	PAO, TIF3E1, JAZ1	1.6056E-3
Downregulated genes			
42221	Response to chemical stimulus	ZAT10, TIP1-1, BCA1, ASN1, TT10,RD21A, LHY, ACC1	9.6473E-5
48608	Reproductive structure development	PAO, NAC29, LHY, ACC1, BXL1	5.0267E-4
9642	Response to light intensity	ZAT10, ASN1	2.1892E-3
9739	Response to gibberellin stimulus	TIP1-1, LHY	4.5114E-3
15979	Photosynthesis	ZAT10, PNSL2	5.0169E-3
32502	Developmental process	UGE3, NAC29, LHY, ACC1 ,BXL1	5.4452E-3
15976	Carbon utilization	BCA1	5.6366E-3

The activity of *PAO* is only seen during senescence; therefore, *PAO* tends to be a central regulator of the catabolism of chlorophyll (chl). The expression of *PAO* interacts favorably with senescence, but the enzyme still tends to be regulated posttranslationally (Pružinská et al., 2003). On the other hand, *TIF3E1* is involved in DNA-templated transcription, flower development and photomorphogenesis. The role of different *TIF3E1* genes has also been investigated in several plants, such as in Arabidopsis (Lim et al., 2013; Yahalom et al., 2008). As reported by Yahalom et al. (2008), *TIF3E1* mutants contribute to diminished fertility and reproductive abnormalities, which tend to cause male gametophyte lethality (Yahalom et al., 2008). Meanwhile, *JAZ1* , which is an essential signaling molecule of the jasmonic acid (JA) pathway, is involved in the reaction to plant pathogens and abiotic stresses (Liu et al., 2017). Other than these stresses, JAZ1 are also known as crucial players in most plant developmental processes, including stamen and flower development (Huang et al., 2017; Zhai et al., 2015). A previous study showed that the MYB transcription factors (TFs) (MYB21 and MYB24) interacted with most JAZ proteins, including JAZ1 where these repressor proteins would interfere with the DNA binding function of MYB21/24. This may attenuate the function of the TFs in regulating the stamen development (Huang et al., 2017). The limited number of *S. rebaudiana* reference genome has made it difficult to establish the actual number of genes involved in the metabolic pathways. In the public databases, a significant number of candidate transcripts may be combined with established enzymes, providing a considerable gene resource for more *S. rebaudiana* analyses. For evolutionary and metabolomic studies, our findings also provided reference sequences among the members of the Asteraceae family which is rich in ethnomedicinal plants. 

## 5. Conclusion

Transcriptomic analysis or RNA-seq based on next-generation sequencing technology is emerging as an attractive approach to understand molecular dynamics of RNA-level interactions. The first detailed transcriptomic profile of *S. rebaudiana* leaf tissues (before and after flowering) with a curation of secondary transcripts linked to metabolites was represented in this report. In prior investigations on specific processes or pathways, particularly the impact of biosynthesis of secondary metabolites, we annotated the assembly data against the existing public databases that provided the established reference transcriptomic profile of *S. rebaudiana* with transcript annotations. We also identified some of the enriched metabolite pathways in the leaf tissues. Based on the analysis of the transcriptome, pheophorbide A oxygenase ( *PAO* ), eukaryotic translation initiation factor 3 subunit E ( *TIF3E1* ), and jasmonate ZIM domain-containing protein 1 ( *JAZ1* ) were found to be involved in the flower development of *S. rebaudiana* MS007. The identification of these genes is related to secondary metabolite biosynthesis, aiding in further exploitations of the genetic resource from this herbal plant for the future biotechnological developments.

## Availability of data and material

The raw sequence data of BF (before flowering sample) and AF (after flowering sample) has been deposited in GenBank under Accession No. SRX6362785 and SRX6362784, respectively (Samsulrizal et al. 2020).

Supplementary Materials

## References

[ref1] (2011). Influence of genetic variation on morphological diversity in accessions of Stevia rebaudiana Bertoni. International Journal of Biology.

[ref2] (1998). Stevia rebaudiana: its agricultural, biological, and chemical properties. Canadian Journal of Plant Science.

[ref3] (2014). Corset: enabling differential gene expression analysis for de novo assembled transcriptomes. Genome Biology.

[ref4] (2012). FLEXBAR-flexible barcode and adapter processing for next-generation sequencing platforms. Biology.

[ref5] (2008). Circadian clock proteins LHY and CCA1 regulate SVP protein accumulation to control flowering in arabidopsis. Plant Cell.

[ref6] (2017). Arabidopsis MYB24 regulates jasmonate-mediated stamen Development. Frontiers in Plant Science.

[ref7] (2014). Effect of two plant growth retardants on steviol glycosides content and antioxidant capacity in Stevia (Stevia rebaudiana Bertoni). Acta Physiologiae Plantarum.

[ref8] (2002). Use of stevioside and cultivation of Stevia rebaudiana in Korea.

[ref9] (2002). Stevia: The Genus Stevia.

[ref10] (2013). The history of diabetes mellitus. Sultan Qaboos University Medical Journal.

[ref11] (2011). RSEM: accurate transcript quantification from RNA-Seq data with or without a reference genome. BMC Bioinformatics.

[ref12] (2013). Arabidopsis PYl8 plays an important role for ABA signaling and drought stress responses. The Plant Pathology Journal.

[ref13] (2017). Plant jasmonate ZIM domain genes: Shedding light on structure and expression patterns of JAZ gene family in sugarcane. BMC Genomics.

[ref14] (2005). BiNGO: a Cytoscape plugin to assess overrepresentation of Gene Ontology categories in biological networks. Bioinformatics.

[ref15] (2015). ClustVis : a web tool for visualizing clustering of multivariate data using Principal Component Analysis and heatmap. Nucleic Acids Research.

[ref16] (2013). Selecting the right similarity-scoring matrix. Current Protocols in Bioinformatics.

[ref17] (2016). Steviol glycosides: natural non-caloric sweeteners. Sweeteners. Cham.

[ref18] (2008). Development of rebiana, a natural, non-caloric sweetener. Food and Chemical Toxicology.

[ref19] (2003). Chlorophyll breakdown: Pheophorbide a oxygenase is a Rieske-type iron-sulfur protein, encoded by the accelerated cell death 1 gene. Proceedings of the National Academy of Sciences of the United States of America.

[ref20] (2012). The Pfam protein families database. Nucleic Acids Research.

[ref21] (1998). Estimation of errors in “Raw” DNA sequences: a validation study. Genome Research.

[ref22] (2001). Specific functions of red, far red, and blue light in flowering and stem extension of long-day plants. Journal of the American Society for Horticultural Science.

[ref23] (2004). Arabidopsis Cys2/His2-type zinc-finger proteins function as transcription repressors under drought, cold, and high-salinity stress conditions. Plant Physiology.

[ref24] (2012). A novel function for a redox-related LEA protein (SAG21/AtLEA5) in root development and biotic stress responses. Plant Cell and Environment.

[ref25] (2020). De novo transcriptome dataset of Stevia rebaudiana accession MS007. Data in Brief.

[ref26] (2018). Stevia leaf to stevia sweetener: exploring ıts science, benefits, and future potential. The Journal of Nutrition.

[ref27] (1997). Pfam: a comprehensive database of protein domain families based on seed alignments. Proteins: Structure, Function and Genetics.

[ref28] (2016). KvLEA, a new ısolated late embryogenesis abundant protein gene from kosteletzkya virginica responding to multiabiotic stresses. BioMed Research International.

[ref29] (2003). The COG database: an updated vesion includes eukaryotes. BMC Bioinformatics.

[ref30] (2004). Gene expression correlation and gene ontology-based similarity: an assessment of quantitative relationships. Proceedings of the 2004 IEEE Symposium on Computational Intelligence in Bioinformatics.

[ref31] (2010). The Arabidopsis eukaryotic translation initiation factor 3, subunit F (AteIF3f), is required for pollen germination and embryogenesis. Plant Journal.

[ref32] (2012). Steviol glycosides from stevia: biosynthesis pathway review and their application in foods and medicine. Critical Reviews in Food Science and Nutrition.

[ref33] (2008). Arabidopsis eIF3e is regulated by the COP9 signalosome and has an impact on development and protein translation. Plant Journal.

[ref34] (2008). Use and misuse of the gene ontology annotations. Nature Reviews Genetics.

[ref35] (2015). Transcriptional mechanism of jasmonate receptor COI1-mediated delay of flowering time in arabidopsis. Plant Cell.

