# Supplementary material for: Transcriptome profiling of Stevia rebaudiana MS007 revealed genes involved in flower development
Source: Turk J Biol. 2021 Jun 23;45(3):314–22. doi: 10.3906/biy-2103-3 (PMC8313940; doi:10.3906/biy-2103-3)
Supplement: Supplementary file 1 — Supplementary Materials [file turkjbio-45-314-sup001.pdf]

Supplementary tables

Table S1. Summary for data quality control of *S. rebaudiana* MS007 raw reads.

Sample	Raw reads	Clean reads	Clean bases	Error (%)	Q20 (%)	Q30 (%)	GC (%)
AF reads	53452080	44277294	6.6G	0.01	97.69	93.85	45.24
BF Reads	62737178	47676660	7.2G	0.01	97.43	93.15	45.06

Table S2. The ratio of annotated genes.

	Number of unigenes	Percentage (%)
Annotated in NR	73959	68.29
Annotated in NT	38099	35.17
Annotated in PFAM	52335	48.32
Annotated in GO	52994	48.93
Annotated in KOG	26800	24.74
Annotated in all databases	12144	11.21
Annotated in at least one database	79708	73.59
Total ınigenes	108299	100

Table S3. Overview of the alignment situation.

Sample name	Total reads	Total mapped
AF	44277294	35636530(80.48%)
BF	47676660	37635756(78.94%)
